# *SHANK2* Mutations Result in Dysregulation of the ERK1/2 Pathway in Human Induced Pluripotent Stem Cells-Derived Neurons and *Shank2*(−/−) Mice

**DOI:** 10.3389/fnmol.2021.773571

**Published:** 2021-11-26

**Authors:** Anne-Kathrin Lutz, Andrea Pérez Arévalo, Valentin Ioannidis, Nadine Stirmlinger, Maria Demestre, Richard Delorme, Thomas Bourgeron, Tobias M. Boeckers

**Affiliations:** ^1^Institute for Anatomy and Cell Biology, Ulm University, Ulm, Germany; ^2^Hôpital Robert-Debré, Paris, France; ^3^Génétique Humaine et Fonctions Cognitives, Institut Pasteur, Université Paris Diderot, Paris, France; ^4^Deutsches Zentrum für Neurodegenerative Erkrankungen (DZNE), Ulm Site, Ulm, Germany

**Keywords:** *SHANK2*, hiPSCs, ERK1/2, neurodevelopment, ASD

## Abstract

SHANK2 (ProSAP1) is a postsynaptic scaffolding protein of excitatory synapses in the central nervous system and implicated in the development of autism spectrum disorders (ASD). Patients with mutations in *SHANK2* show autism-like behaviors, developmental delay, and intellectual disability. We generated human induced pluripotent stem cells (hiPSC) from a patient carrying a heterozygous deletion of *SHANK2* and from the unaffected parents. In patient hiPSCs and derived neurons *SHANK2* mRNA and protein expression was reduced. During neuronal maturation, a reduction in growth cone size and a transient increase in neuronal soma size were observed. Neuronal proliferation was increased, and apoptosis was decreased in young and mature neurons. Additionally, mature patient hiPSC-derived neurons showed dysregulated excitatory signaling and a decrease of a broad range of signaling molecules of the ERK-MAP kinase pathway. These findings could be confirmed in brain samples from *Shank2*(−/−) mice, which also showed decreased mGluR5 and phospho-ERK1/2 expression. Our study broadens the current knowledge of SHANK2-related ASD. We highlight the importance of excitatory-inhibitory balance and mGluR5 dysregulation with disturbed downstream ERK1/2 signaling in ASD, which provides possible future therapeutic strategies for SHANK2-related ASD.

## Introduction

SHANK proteins (SHANK1, SHANK2/ProSAP1, and SHANK3/ProSAP2) (Boeckers et al., 1999, 2002; Sheng and Kim, 2000; Grabrucker et al., 2011) function as postsynaptic anchoring platforms for receptor complexes and signaling molecules (Tu et al., 1999; Boeckers et al., 2001; Roussignol et al., 2005) and are involved in synapse formation and modulation (Sala et al., 2001; Boeckers et al., 2005). Genetic variations in all three *SHANK* gene loci have been linked to autism spectrum disorders (ASDs) (Durand et al., 2007; Moessner et al., 2007; Bourgeron, 2009; Berkel et al., 2010; Sato et al., 2012; Huguet et al., 2013). ASDs are neurodevelopmental disorders that manifest in core symptoms (social communication deficits and stereotypic repetitive behavior) (World Health Organization [WHO], 2004; DSM-V, and Centers for Disease Control and Prevention [CDC], 2009) and a broad range of comorbidities.

In *Shank2* deletion mouse models, anxiety-like behavior, hyperactivity, and abnormal social behavior have been observed accompanied by a brain region-specific dysregulation of synaptic molecules, including receptors (especially NMDA receptors), cell adhesion proteins, and members of various signaling cascades (Schmeisser et al., 2012; Won et al., 2012). Excitatory-inhibitory balance (E-I-balance) is regarded as a critical core mechanism in ASD (Zoghbi and Bear, 2012; Lee et al., 2017). Different ASD models show changes in E-I balance (Dickinson et al., 2016), and studies on human mid-fetal circuits showed that the deformation of connectivity and neuronal networks emerge already early in neurodevelopment (Parikshak et al., 2013; Willsey et al., 2013). Other hypotheses concentrate on the critical role of dysregulated mRNA translation (Monteiro and Feng, 2017; Joo and Benavides, 2021) or signaling pathways (Zoghbi and Bear, 2012; Kleijer et al., 2014). In this respect, the extracellular signal-regulated kinase (ERK) pathway seems to play a central role in the pathogenesis of ASD (Faridar et al., 2014; Vithayathil et al., 2018). Many of the genes implicated in ASD are known to interact with components of the ERK pathway (Rosina et al., 2019; Wang et al., 2020). Syndromic forms of ASD, including Rett syndrome and Fragile X syndrome, show dysregulation of the ERK-signaling cascade (Vithayathil et al., 2018).

Extracellular signal-regulated kinase, together with Jun kinase and p38 MAP kinases, builds up the family of mitogen-activated protein kinases (MAPK). Growth factors or cytokines activate a consecutive series of at least three kinases that integrate the extracellular signals and provoke an appropriate cellular response, including proliferation, differentiation, development, inflammation, and apoptosis (Zhang and Liu, 2002). The downstream effectors of the MAPK-signaling cascades are both cytosolic and nuclear proteins, further kinases, and transcription factors as, for example, cAMP response element-binding protein (CREB) (Tidyman and Rauen, 2009). Given these fundamental roles in cellular maintenance, disturbance of the MAPK pathway during neurodevelopment results in inappropriate neuronal maturation and synaptogenesis (Vithayathil et al., 2018).

Comparing patient-derived and non-affected cell lines, disturbed signaling pathways, but also altered regulation of early cellular differentiation and synaptogenesis, can ideally be studied in human induced pluripotent stem cells (hiPSCs). Tremendous progress in development of morphogen and growth factor-based differentiation protocols has been made, allowing the analysis of neuronal precursors and mature human neurons. Various ASD genes have already been studied in hiPSC-derived differentiated cells (Pasca et al., 2011; Kumari et al., 2015; Patriarchi et al., 2016), including *SHANK3* (Shcheglovitov et al., 2013; Bidinosti et al., 2016; Darville et al., 2016; Kathuria et al., 2017) and *SHANK2* (Zaslavsky et al., 2019; Chen et al., 2020). We generated hiPSC-derived neurons from a family consisting of a healthy father and a mother and a son with a *de novo* heterozygous *SHANK2* deletion of 1.8 Mb on chr.11q13.3q13.4 published previously (Leblond et al., 2014). The patient manifests with autism, global developmental delay, severe intellectual disability, non-verbalism, slight hypotonia, and several dysmorphic signs (Leblond et al., 2014). In this study, we analyzed cellular and molecular mechanisms associated with this *SHANK2* deletion in hiPSCs, young and mature hiPSC-derived neurons in comparison to two healthy cell lines.

Our human *in vitro* model system gives evidence that *SHANK2* deletion impairs the neurodevelopment and the synaptic composition in hiPSC-derived neurons. We found major components of the extracellular signal-regulated kinase 1/2 (ERK1/2) pathway to be dysregulated and confirmed these findings in a *Shank2* deletion mouse model.

## Materials and Methods

### Animals

All animal experiments were performed in compliance with the guidelines for the welfare of experimental animals issued by the Federal Government of Germany, the National Institute of Health or the Max Planck Society. The experiments in this study were approved by the review board of the Land Baden-Württemberg, permit numbers 0.103, 321/16 and 966/2016-PR, respectively. *Shank2*(−/−) mice (*mus musculus*) were generated as previously described (Schmeisser et al., 2012). Breeding was carried out as heterozygous breeding on a C57BL/6 background; animals were housed in standard laboratoryconditions (average temperature of 22°C with food and water available *ad libitum*, dark/light cycle as 12/12 rhythm).

### Generation and Maintenance of Human Induced Pluripotent Stem Cells

Patient and control hiPSCs were reprogrammed from hair keratinocytes, following a published protocol (Aasen et al., 2008; Stockmann et al., 2013). Hair was obtained from Thomas Bourgeron, Paris, France. hiPSCs were grown on hESC-qualified matrigel (BD Biosciences) in mTeSR1 medium (Stemcell Technologies) at 37°C, 5% O_2_, and 5% CO_2_.

### Germline Differentiation and Karyotyping

Human induced pluripotent stem cells were spontaneously differentiated in all three germlines by formation of embryoid bodies (EBs) in hESC medium (DMEM/F12 + GlutaMAX, 20% knockout serum replacement, 1% NEAA, 1% antibiotic-antimycotic [all Gibco), 1% β-mercaptoethanol (Millipore)] in low-attachment flasks. ROCK inhibitor was added the first 24 h. Cells were kept 7 days as free-floating EBs, plated on 35 mm μ-dishes (Ibidi) coated with poly-L-ornithine and laminin and grown further for 21 days. Cells were fixed and stained for ectoderm, mesoderm, and endoderm using the following primary antibodies: β-III-TUBULIN (chicken, 1:1,000, Millipore), α-Actinin (mouse, 1:500, Sigma-Aldrich) and alpha-fetoprotein (AFP, goat, 1:100, Santa-Cruz). hiPS cell lines were karyotyped to exclude cell lines-harboring chromosomal aberrations after reprogramming (Linta et al., 2012). hiPSCs were treated with 1.5 M Colchicine (Eurobio) diluted in mTeSR1 for 2 h at 37°C. Cells were trypsinized (TrypLE, Gibco), and metaphases were kindly analyzed by the Institute for Human Genetics, Ulm University.

### Neuronal Differentiation of Human Induced Pluripotent Stem Cells

Neural differentiation followed previously published protocols (Hu and Zhang, 2009; Stockmann et al., 2013). hiPSCs were lifted using dispase (Stemcell Technologies) and grown as EBs in low-attachment flasks (Corning Costar) using hESC medium. ROCK inhibitor (Ascent Scientific) was added the first 24 h. Medium was changed to neuronal basal-medium [DMEM/F12, 0.02 mg/ml insulin (SAFC), 24 nM sodium selenite, 16 nM progesterone, 0.08 mg/ml apotransferrin, 7.72 μg/ml putrescin, 50 mg/ml heparin (all Sigma-Aldrich), 1% NEAA, 1% antibiotic-antimycotic, 10 μg/ml BDNF, GDNF, and IGF-1 (all Gibco), 0.1 μMcAMP (Sigma-Aldrich), 50 mg/ml ascorbic acid (PeproTech)] at Day 3 of culture. At Day 7, EBs were plated on laminin (Sigma)-coated dishes. 0.1 μM retinoic acid was added from Day10 and B27 (Gibco) and 1 μM purmorphamine (Calbiochem) from Day 14, when neural rosettes were detached and grown in suspension again. At Day 28, EBs were plated on 35 mm μ-dish (Ibidi) coated with poly-L-ornithine (Sigma) and laminin. The medium was changed to 0.5 μM purmorphamine and 0.05 μM retinoic acid and changed one time per week. Neuronal cells were harvested or fixated at Days 21 and 42 after final plating.

### Real-Time qRT-PCR

Total RNA was isolated using the RNeasy Mini Kit (Qiagen), following the instructions of the manufacturers. Cell pellets were lysed in an RLT buffer. Quantitative real-time PCR was carried out using the one-step QuantiFast SYBR Green RT-PCR kit (Qiagen) and a Rotor-Gene-Q real-time PCR machine (Model 2-Plex HRM, Qiagen). All primers were obtained from Qiagen (QuantiTect Primer Assay). Amplification was carried out under the following conditions: 10 min at 55°C and 5 min at 95°C, followed by 40 cycles of 5 s at 95°C and 10 s at 60°C (one-step qRT-PCR). Rotor-Gene-Q software (version 2.0.2) calculated the cycle threshold (Ct) values. All measurements were run in technical duplicates and normalized to housekeeping genes hydroxymethylbilane synthase (HMBS) or neurofilament H (NFH).

### Immunoblotting

Cell pellets were lysed in a RIPA buffer [150 mM NaCl (Sigma); 50 mM Tris/HCl (AppliChem); 1% NP-40; 0.5% Sodium deoxycholate (Merck); 0.1% SDS; Protease inhibitor (Roche); and Phosphatase inhibitor (Roche)] for 45 min on ice, sonicated (10 pulses), and cleared for 10 min at 13.000 rpm. Mouse brain tissue was homogenized in a lysis buffer (10 mM HEPES pH 7.4, 2 mM EDTA, 5 mM Sodium Orthovanadate, 30 mM Sodium Fluoride, 20 mM β-glycerol phosphate, complete (Roche). Protein concentration was quantified using a colorimetric measurement of protein in Bradford solution [0.1% Serva Blue Powder (Serva), 8.5% Phosphoric acid (VWR), 4.75% ethanol in H_2_O]. Equal protein amount was boiled in an SDS-loading buffer [200 mMTris/HCl (pH6.8); 200 mM DTT (Sigma); 4% SDS (Roth); 4 mM EDTA (AppliChem); 40% Glycerol (AppliChem); 0.2% Bromphenol blue (Sigma)] and separated using SDS-PAGE. Blotting on nitrocellulose membranes was performed using Trans-Blot^®^ Turbo^TM^ RTA Midi Nitrocellulose Transfer Kit (BioRAD, 1704271). Membranes were blocked in 5% BSA (AppliChem) or 5% milk powder (Fluka) in 0.1% TBS-Tween 20 and incubated with primary antibodies. HRP-conjugated secondary antibodies were visualized with ECL Western Blot substrate (Pierce) and a MicroChemi 4.2 machine. Signals were quantified using Gelanalyzer Software and normalized against the corresponding loading control. The following primary antibodies were used: SHANK2 “ppI-SAMpabSA5192” (rabbit, 1:000 (Boeckers et al., 1999; Schmeisser et al., 2012), SHANK3/ProSAP2 (Fragment 1 + 2, Tier 2, rabbit, 1:500 (Schmeisser et al., 2012), mGluR5 (rabbit, 1:500, Abcam), vGLUT1 (guinea pig, 1:500, SYSY), GluA2 (mouse, 1:500, SYSY), GAD65 (mouse, 1:500, abcam ab26113), GABA_B_R1 (rabbit, 1:200, SYSY), ERK1/2 (rb, 1:1,000, Cell Signaling, 4695), phospho-ERK1/2 (Thr202/Tyr204, rb, 1:2,000, Cell Signaling 4270), Akt (rb, 1:1,000, Cell Signaling 4691), phospho-AKT (Thr308, rb, 1:1,000, Cell Signaling 13038), NFH (mouse, 1:1,000, Convance), β-actin (mouse, 1:100,000, Sigma).

### Cleaved Caspase3 Assay Kit

Caspase 3 Assay Kit (Colorimetric) was purchased from Abcam ab39401. Measurements were carried out following the instructions of the manufacturers. Cells were lysed in 50 μL of chilled Cell Lysis Buffer. Protein concentration was assessed by a Bradford assay as previously described. About 50 μg per 50 μL protein was measured after 1 h incubation at 37°C for 60 min. Output was measured at 405 nm on a Cytation^TM^ 3 Cell Imaging Multi-Mode Reader (BioTek).

### Immunocytochemistry

Cells were fixed using 4% paraformaldehyde (Merck)/4% sucrose (Roth) in PBS at 37°C for 15 min and washed three times with PBS + Ca^2+^/Mg^2+^ (PAA). Fixed cells were permeabilized with 0.2% Triton-X100 (Roche) in PBS for 10 min and blocked in 5% fetal bovine serum (Gibco)/10% goat serum (Millipore) in PBS for at least 2 h. Primary antibodies were incubated at 4°C for 48 h in blocking solution. After three-time wash with PBS, secondary antibodies coupled to Alexa Fluor 488, 568, or 647 (Life Technologies) were incubated for 1 h at RT in blocking solution. Cells were mounted with ProLong^®^ Gold antifade reagent with DAPI (Thermo Fisher Scientific). The following primary antibodies were used: SHANK2 “ppI-SAMpabSA5192” [rabbit, 1:500, (Boeckers et al., 1999; Schmeisser et al., 2012)], SHANK3/ProSAP2 [Fragment 1 + 2, Tier 2, rabbit, 1:500 (Schmeisser et al., 2012)], Ki67 (rabbit, 1:500, Abcam), Active Caspase3 (rabbit, 1:500, R&D Systems), GluA2 (mouse, 1:500, SYSY), Bassoon (mouse, 1:500, SYSY), mGluR5 (rabbit, 1:500, Abcam), vGLUT1 (guinea pig, 1:500, SYSY), vGAT (rabbit, 1:500, SYSY), GABA_*A*_Rα1 (mouse, 1:200, NeuroMab), Homer1 (guinea pig, 1:500, SYSY), GluN1 (rabbit, 1:500 Sigma), ERK1/2 (rb, 1:200, Cell Signaling 4695), phospho-ERK1/2 (Thr202/Tyr204, rb, 1:1,000, Cell Signaling 4270), phospho-CREB (S133, rb:1:500, Abcam 32096), MAP2 (ck, 1:1,000, EnCor), NFH (chicken, 1:50,000, Antikörper online), and StemLite Pluripotency Kit (Cell Signaling, #9656). Fluorescent images were recorded using an Axioscope microscope with a Zeiss CCD camera (16 bits; 1,280 × 1,024 ppi) and Axiovision software (Zeiss).

### Full Moon Assay

The protein array [Full Moon Biosystems, ERK phospho Antibody Array (PEK208)] was performed according to the instructions of the manufacturer using three replicates per cell line and time point. Briefly, neurons were lysed in a 100 μl extraction buffer (Biosystems) supplemented with protease and phosphatase inhibitors (Roche). Protein concentration was determined performing and A280 assay. Blocking and coupling were performed according to the protocol of the manufacturer. Biotinylated samples were labeled with Cy5 Streptavidin (Invitrogen SA1011). The detection of antibody arrays was performed in a fluorescent slide scanner (Genepix 4000B microarray scanner, Molecular Devices). The 16-bit images were analyzed using the GenePix Pro 6.1 software. The data had to fulfill the requirements of a signal-noise ratio (SNR) above 3 in the 635 channels, and all flags needed to be zero. For quantification, the mean of six technical replicates per antibody was used and set relative to β-ACTIN.

R version 4.1.0 was used for all the following steps of the analysis. Data were grouped consecutively by batch, timepoint, and protein. Subgroups were normalized, respectively, on the mean of maternal and paternal measurements to return fold change values (FC). Pheatmap R package was used to create heatmaps and perform hierarchical clustering using Euclidean distance with a “complete” method. The heatmaps show Z-scored FC per protein. For labeled heatmaps, data were filtered for proteins with FC of either 0.45 greater or smaller for both a mother and a father in comparison to a patient, revealing changes in the patient between 0.65 and 1.45 relative to the mean of the parents. The FC was calculated using the following formula: [father < (patient - fc.threshold) and mother < (patient - fc.threshold)] or [father > (patient + fc.threshold) and mother > (patient + fc.threshold)].

### Image Analysis and Quantification

Fluorescent images were analyzed using Axiovision and ImageJ software. For synaptic analysis, pictures were deconvoluted using Autoquant X3 Deconvolution software (Imaris). Intensity of foci along neurites was measured using the “Find Foci” plugin of ImageJ (Herbert et al., 2014) as previously published (Higelin et al., 2016). Neuronal soma size was measured manually in the NFH immunostaining channel using ImageJ. For analysis of growth cones size, a polygonal region of interest containing only one growth cone was selected using ImageJ. A threshold was set for the GAP-43 staining, and the respective size of the positive area was obtained. For analysis of Ki67 positive cells, the number of NFH and DAPI-positive neurons per picture was counted and set to 100%. Then, Ki67 positive nuclei in NFH-positive neurons were counted, and the percentage was calculated. Per experiment and cell line, nine pictures were analyzed. For analysis of ERK1/2 and phospho-ERK1/2 intensity analysis, the soma of MAP2 positive neurons was surrounded manually, and the mean gray value of ERK1/2 or phospho-ERK1/2 was measured in the selected area. For analysis of phospho-CREB intensity, the DAPI signal of MAP2 positive neurons was surrounded manually, and the intensity of phospho-CREB signal was measured within the selection.

### Statistical Analysis

Statistical analysis was performed using GraphPad Prism 6. Significance value was set to 0.05 with ^∗^*p* ≤ 0.05, ^∗∗^*p* ≤ 0.01, ^∗∗∗^*p* ≤ 0.001, and ^****^p ≤ 0.0001. Values were tested using Student‘s *t*-test or one-way ANOVA, followed by *post hoc* analysis (Tukey’s multiple comparison test) as indicated.

## Results

### SHANK2 Levels Are Decreased in Human Induced Pluripotent Stem Cells and Derived Neurons of a *SHANK2* Deletion Patient

**FIGURE 1:**
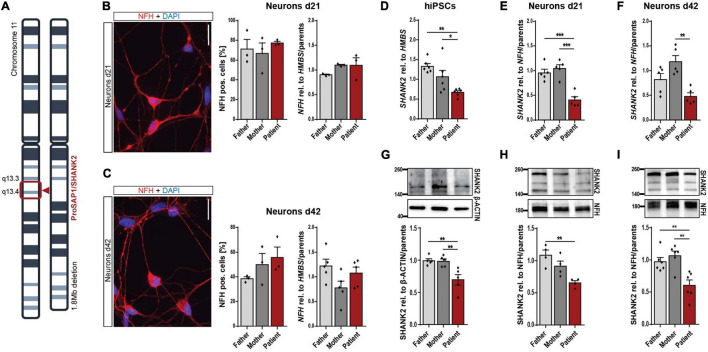
Neuronal differentiation and reduction of SHANK2 expression in hiPSCs and derived neurons. **(A)** Chromosomal deletion of the patient with *SHANK2* deletion. **(B,C)** Neurons d21 **(B)** and d42 **(C)** stained for NFH and DAPI. Percentage of NFH-positive cells (*n* = 3). *NFH* RNA levels in total cell lysate (d21 *n* = 3; d42 *n* = 5). **(D)** *SHANK2* RNA relative to *HMBS* (*n* = 6) in hiPSCs. **(E)** *SHANK2* RNA relative to *NFH* (*n* = 5) in neurons d21. **(F)** *SHANK2* RNA relative to *NFH* (*n* = 5) in neurons d42. **(G)** SHANK2 protein expression relative to β-ACTIN (*n* = 5) in hiPSCs. **(H)** SHANK2 protein levels relative to NFH (*n* = 4) in neurons d21. **(I)** SHANK2 protein levels relative to NFH (*n* = 6) in neurons d42. NFH, neurofilament heavy chain; HMBS, hydroxymethylbilane synthase. Scale bar = 20 μm. Data were normalized to mean of parents if indicated. Mean ± SEM, one-way ANOVA, followed by Tukey’s *post hoc* analysis. Significance levels were set to *p ≤ 0.05, **p ≤ 0.01, and ***p ≤ 0.001.

The *SHANK2* deletion patient analyzed in this study carries a heterozygous deletion of 1.8 Mb on chromosome 11, comprising *SHANK2* (Figure 1A). Keratinocytes grown from hair roots of the patient, the father, and the mother were reprogrammed to hiPSCs, following previously published protocols (Supplementary Figure 1A and Linta et al., 2012). Generated hiPSCs expressed pluripotency markers SOX2, OCT4, NANOG, TRA-1-81, TRA-1-60, and SSEA4 in immunostaining (Supplementary Figure 1B) and SOX2, OCT4, NANOG, and KLF4 on the RNA level (Supplementary Figure 1C). They differentiated spontaneously into all three germlines as shown by immunostaining for α-ACTININ (mesoderm), alpha-fetoprotein (AFP, endoderm), and β-III-TUBULIN (ectoderm) (Supplementary Figure 1D). No genetic alterations were detectable by karyogram analysis in all three cell lines (Supplementary Figure 1E). We differentiated hiPSCs into neurons following previously established protocols (Hu and Zhang, 2009; Stockmann et al., 2013) and analyzed young neurons after 21 days (d21) and mature neurons after 42 days (d42) of final plating. Patient and parent neuronal cultures contained comparable numbers of neurofilament heavy chain (NFH)-positive neurons and equal *NFH* levels relative to housekeeping gene *HMBS* in d21 (Figure 1B) and d42 cultures (Figure 1C). About 40 to 50% of cells in mature d42 cultures were found to be NFH-expressing neurons, as previously reported for this protocol (Stockmann et al., 2013; Higelin et al., 2018). *SHANK2* RNA levels were significantly decreased to approximately 50% in hiPSCs (Figure 1D), neurons d21 (Figure 1E), and neurons d42 (Figure 1F). Analysis of SHANK2 protein expression revealed similar reductions in hiPSCs (Figure 1G), neurons d21 (Figure 1H), and neurons d42 (Figure 1I), indicating haploinsufficiency in heterozygously *SHANK2*-deleted cells. SHANK2 localization in patient hiPSCs was not altered (Supplementary Figure 2A).

### Neuronal Development and Maturation Are Impaired in *SHANK2* Deletion Human Induced Pluripotent Stem Cells-Derived Neurons

**FIGURE 2:**
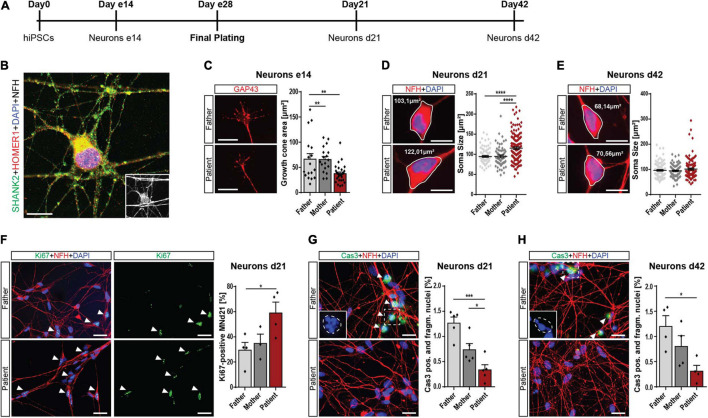
Neurodevelopmental changes in *SHANK2* deletion neurons. **(A)** The differentiation scheme of hiPSC-derived neurons. **(B)** A 42-day-old neuron stained against SHANK2, HOMER1, and NFH shows a mature morphology and synaptic co-localization. Inlay: NFH. Scale bar = 10 μm. **(C)** Staining for GAP43 in e14 neurons. The growth cone area was quantified. Data collected form three independent experiments. Scale bar = 20 μm. **(D,E)** Staining for NFH. Soma size [μm^2^] of NFH-positive neurons was measured in neurons d21 **(D)** and d42 **(E)** Scale bar = 10 μm. **(F)** Staining of neurons d21 for Ki67 and NFH. Percentage of Ki67-positive neurons was quantified. Data collected from four independent experiments. Scale bar = 10 μm. **(G,H)** Staining for cleaved caspase 3 and NFH. Neurons positive for cleaved caspase 3, and manifesting fragmented nuclei were assessed. *n* = 5 for d21 **(G)** and *n* = 4 for d42 **(H)**. Inlay shows fragmented nuclei. The estimated shape of the original non-fragmented nucleus is illustrated. Scale bar = 20 μm. NFH, neurofilament heavy chain. Data were normalized to mean of parents if indicated. Mean ± SEM, one-way ANOVA, followed by Tukey’s *post hoc* analysis. Significance levels were set *p ≤ 0.05, **p ≤ 0.01, ***p ≤ 0.001, and ****p ≤ 0.0001.

During neuronal differentiation from hiPSCs, the cells pass various stages summarized in Figure 2A. Forty-two days after plating, they reached a mature morphology characterized by expression of synaptic proteins, including SHANK2 and HOMER1 (Figure 2B). To address the early neuronal morphology, we analyzed the growth cone area in early 14-day-old cells (e14) and found reduced sizes in patient cells (Figure 2C), indicating an early developmental delay. Furthermore, the soma size was significantly increased in d21 patient cells, but not in d42 cells (Figures 2D,E). The number of primary neurites was not altered in patient d21 or d42 neurons (Supplementary Figures 2B,C). These changes during early neuronal development went along with significantly increased cell proliferation in these d21 neurons (Figure 2F). In addition, we found significantly decreased percentages of neurons with fragmented nuclei positive for cleaved caspase 3 in d21 (Figure 2G) and d42 neurons (Figure 2H). Assessment of cleaved caspase 3 levels in total protein lysate in a colorimetric assay showed the same trend (Supplementary Figure 2D). Thus, we conclude that *SHANK2* deletion correlates with increased soma size, increased proliferation, and decreased apoptosis in immature d21 neurons during development. Decreased apoptosis seems to be a persistent phenotype that is also observed in mature d42 neurons.

### Synaptic SHANK2 Levels Are Decreased, and SHANK3 Levels Increased in the *SHANK2* Deletion Patient

**FIGURE 3:**
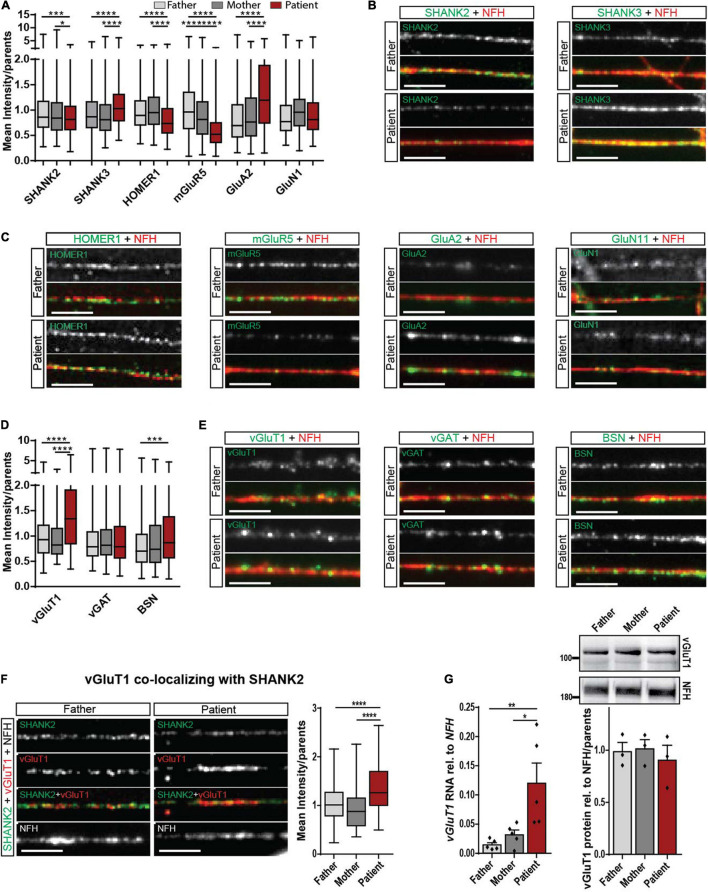
Synaptic alteration in the patient with *SHANK2* deletion in neurons d42. **(A)** Analysis of mean synaptic puncta intensity for SHANK2, SHANK3, HOMER1, mGluR5, GluA2, and GluN1. Data were normalized to mean of parents. **(B)** Representative staining for SHANK2, SHANK3, and NFH. Scale bar = 5 μm. **(C)** Representative staining for HOMER1, mGluR5, GluA2, GluN1, and NFH. Scale bar = 5 μm. **(D)** Analysis of mean synaptic puncta intensity for vGluT1, vGAT, and BSN. Data were normalized to mean of parents. **(E)** Representative staining for vGluT1, vGAT, BSN, and NFH. Scale bar = 5 μm. **(F)** Staining for SHANK2, vGluT1, and NFH. Analysis of the mean intensity of vGluT1 puncta co-localizing with SHANK2. Data were normalized to mean of parents. **(G)** *vGluT1* RNA relative to *NFH* (*n* = 6) and vGluT1 protein levels relative to NFH (*n* = 3) in neurons d42. vGluT1, vesicular glutamate transporter 1; vGAT, vesicular GABA transporter; NFH, neurofilament heavy chain; BSN, bassoon; mGluR5, metabotropic glutamate receptor 5; GluA2, AMPA receptor subunit 2; GluN1, NMDA receptor subunit 1. Box plots show the median and the distribution of the minimum to the maximum data. One-way ANOVA followed by Tukey’s *post hoc* analysis. Significance levels were set to *p ≤ 0.05, **p ≤ 0.01, ***p ≤ 0.001 and ****p ≤ 0.0001.

Regarding the important role of SHANK molecules at synapses, we analyzed SHANK2 and SHANK3 expression directly at the synapse. The mean intensity of synaptic SHANK2 puncta along neurites was significantly decreased (Figures 3A,B), while the intensity of synaptic SHANK3 puncta was significantly increased in patient-derived cells (Figures 3A,B). No alterations of SHANK3 were found in cellular localization in hiPSCs (Supplementary Figure 2E) and in RNA and protein expression of total cell lysate (Supplementary Figures 2F–H). These data indicate a possible synaptic compensation of SHANK3 for the lacking SHANK2 protein.

### Glutamatergic Signaling Is Dysregulated in *SHANK2* Deletion Human Induced Pluripotent Stem Cells-Derived Neurons

To obtain a complete picture of glutamatergic signaling, we assessed pre- and postsynaptic proteins in d42 neurons. For the postsynaptic specialization, we analyzed HOMER1 as an interaction partner of both SHANK2 and SHANK3 and mGluR5 that is included in critical pathways contributing to ASD (Bear et al., 2004; Vicidomini et al., 2017). As reported previously, synaptic HOMER1 decreased (Vicidomini et al., 2017). We found decreased mGluR5 levels and increased synaptic AMPA receptor subunit GluA2 levels (Figures 3A,C), but no changes in GluN1 levels in *SHANK2* deletion neurons. Assessment of the total protein intensity for all synaptic stainings revealed the same alterations between parents and patients compared with the mean intensity (Supplementary Figure 3A), stressing that, indeed, the protein concentrations are altered. Regarding the number of puncta per 30 μm of dendrite, mGluR5 was the only protein showing a decrease in the patient cells (Supplementary Figure 3B), suggesting a critical dysregulation of mGluR5.

In the pre-synapse, glutamate reuptake into synaptic vesicles is mediated by vesicular glutamate transporter 1 (vGluT1). We found a significantly increased mean intensity of vGLUT1 immunostaining in SHANK2 patient neurons (Figures 3D,E), while levels of vesicular GABA transporter (vGAT), the respective transporter at inhibitory synapses, were unchanged (Figures 3D,E). Total protein intensity revealed the same results (Supplementary Figure 3C), and the number of puncta per 30 μm was not changed (Supplementary Figure 3D). Indeed, vGluT1 levels were also increased in synapses, co-expressing SHANK2 and vGluT1, as shown by co-localization analysis of the two proteins (Figure 3F and Supplementary Figures 3E–G). In addition, increased vGluT1 levels were found in total RNA, but not in protein lysate (Figure 3G), indicating a specific neuronal synaptic alteration. Immunostaining for bassoon (BSN), a presynaptic scaffolding protein, revealed increased BSN levels in SHANK2 patient neurons (Figures 3D,E), indicating complex presynaptic alterations. However, protein levels in total cell lysate showed no changes between parents and patients (Supplementary Figure 3H), revealing the synapse as a critical point for dysregulation in SHANK2-related ASD.

### *SHANK2* Deletion Affects Extracellular Signal-Regulated Kinase 1/2 Signaling in Human Induced Pluripotent Stem Cells-Derived Neurons

**FIGURE 4:**
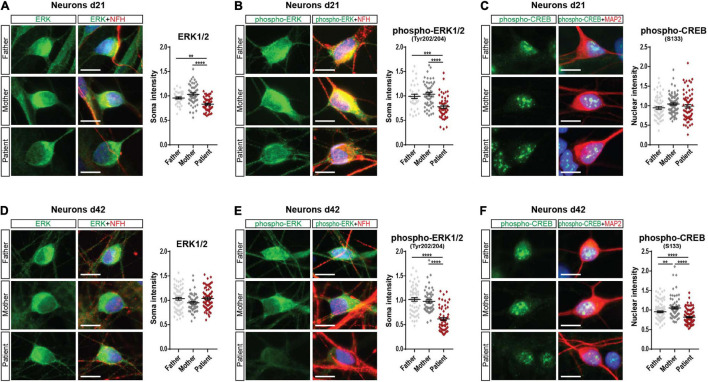
Phospho-ERK1/2 and phospho-CREB are decreased in the *SHANK2* deletion patient. **(A)** Staining of neurons d21 for ERK1/2 and NFH. The soma intensity of ERK1/2 was quantified. Data were collected from three independent cultures per cell line. Number of cells F, *n* = 37; M, *n* = 50; P, *n* = 51. **(B)** Staining of neurons d21 for phospho-ERK1/2 and NFH. The soma intensity of phospho-ERK1/2 was quantified. Data were collected from three independent cultures per cell line. Number of cells F, *n* = 35; M, *n* = 50; P, *n* = 47. **(C)** Staining of neurons d21 for phospho-CREB and MAP2. The soma intensity of phospho-CREB was quantified. Data were collected from three independent cultures per cell line. Number of cells F, *n* = 64; M, *n* = 79; P, *n* = 67. **(D)** Staining of neurons d42 for ERK1/2 and NFH. The soma intensity of ERK1/2 was quantified. Data were collected from three independent cultures per cell line. Number of cells F, *n* = 70; M, *n* = 50; P, *n* = 64. **(E)** Staining of neurons d42 for phospho-ERK1/2 and NFH. The soma intensity of phospho-ERK1/2 was quantified. Data were collected from three independent cultures per cell line. Number of cells F, *n* = 65; M, *n* = 53; P, *n* = 57. **(F)** Staining of neurons d42 for phospho-CREB and MAP2. The soma intensity of phospho-CREB was quantified. Data were collected from three independent cultures per cell line. Number of cells F, *n* = 101; M, *n* = 80; P, *n* = 94. NFH, neurofilament heavy chain; MAP2, microtubule-associated protein 2. Scale bar = 10 μm. Data were normalized to mean of parents. Mean ± SEM, one-way ANOVA, followed by Tukey’s *post hoc* analysis. Significance levels were set to **p ≤ 0.01, ***p ≤ 0.001, and ****p ≤ 0.0001.

Since we found decreased mGluR5 levels in *SHANK2*-deleted hiPSC-derived neurons, we analyzed extracellular signal-regulated kinase 1/2 (ERK1/2) and AKT as main mGluR5 downstream-signaling pathways. In Western Blot analysis of total protein lysate, we did not observe any changes in d21 neurons (Supplementary Figures 4A,B). In d42 neurons, ERK1/2 and phospho-ERK1/2 were significantly decreased (Supplementary Figure 4C) but not AKT or phospho-AKT expression (Supplementary Figure 4D). Western Blot analysis for CREB and phospho-CREB, a transcription factor downstream of the ERK-signaling cascade, did not show any changes in d21 or d42 (Supplementary Figures 4E,F). To analyze neurons only, we performed immunostainings and analyzed ERK1/2, phospho-ERK1/2, and phospho-CREB in NFH and MAP2 positive neurons. Indeed, d21 neurons showed decreased ERK1/2 (Figure 4A) and phospho-ERK1/2 (Figure 4B) expression. Phospho-CREB was not altered (Figure 4C). d42 neurons showed unchanged neuronal ERK1/2 expression (Figure 4D), but both phosphorylated ERK1/2, the active version of ERK1/2 (Figure 4E), and phospho-CREB (Figure 4F) were significantly lower in the patient, speaking for a differential activation of the ERK-signaling cascade under *SHANK2* deletion.

### *SHANK2* Deletion Affects Multiple Mediators of Extracellular Signal-Regulated Kinase 1/2 Signaling

**FIGURE 5:**
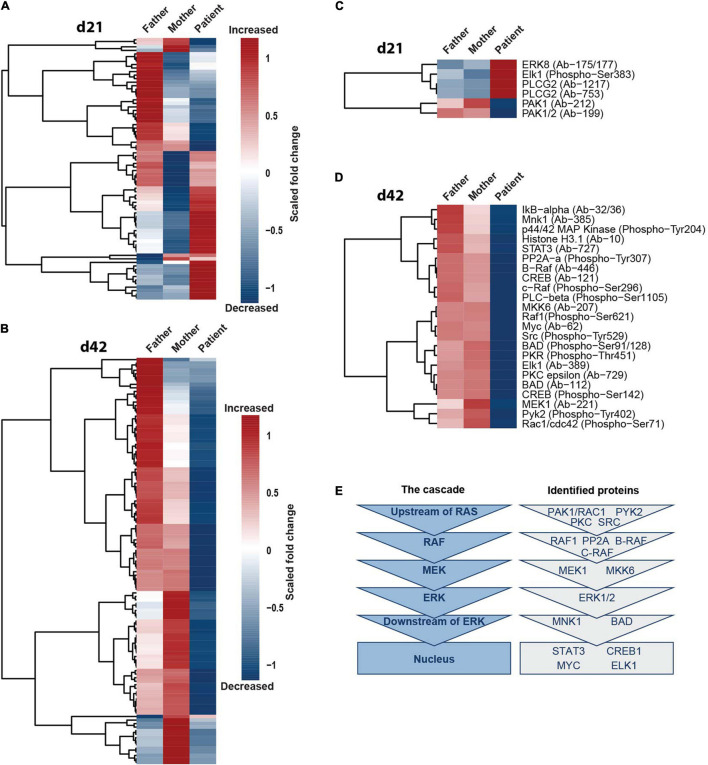
*SHANK2* deletion affects the ERK1/2 signaling pathway in general. **(A,B)** Full Moon array with neurons d21 **(A)** and d42 **(B)**. Scored fold change to the mean of maternal and paternal measurements per protein is shown, *n* = 3. **(C,D)** Neurons d21 **(C)** and neurons d42 **(D)**. Data were filtered for proteins with a fold change of either 0.45 greater or smaller for both a mother and a father in comparison to the patient. **(E)** Attributing the proteins filtered in **(C,D)** to the six main hierarchies in the ERK1/2 signaling pathway upstream of RAS, RAF, MEK, ERK, downstream of ERK, and in the nucleus.

To investigate if only ERK1/2 and phospho-CREB or also additional components of the MAPK-signaling cascade are affected by SHANK2 loss, we performed protein arrays, targeting the ERK1/2 pathway in d21 and d42 hiPSC-derived neurons of father, mother and patient. In d21 neurons, the data derived from the patient clustered differently than from the father and the mother, showing both up- and downregulated proteins in comparison to the two parents (Figure 5A). In d42 neurons, the changes in the patient in comparison to the father and the mother were even more obvious, since almost all proteins analyzed showed a drastically lower expression in the patient (Figure 5B). To identify proteins distinctly up- or downregulated between the patient and parents, we filtered only for those proteins that showed a fold change of 0.45 greater or smaller for the patient in comparison to both the mother and the father. In d21 neurons, we obtained a group of four up- and two downregulated proteins (Figure 5C), and, in d42 neurons, all proteins were downregulated, including phospho-ERK1/2 (p44/42 MAP kinase phospho-Tyr204) and phospho-CREB (Figure 5D) that we analyzed before. In the ERK1/2 pathway, a series of kinases is activated by consecutive phosphorylation, leading to the activation of a variety of transcription factors (Molina and Adjei, 2006). The identified proteins represented all levels of the ERK1/2-signaling hierarchy (Figure 5E), supporting our hypothesis of a general dysregulation of the ERK1/2-signaling cascade under *SHANK2* deletion.

### Extracellular Signal-Regulated Kinase 1/2 Signaling Is Altered in Young but Not in Old *Shank2(−/−)* Mice

**FIGURE 6:**
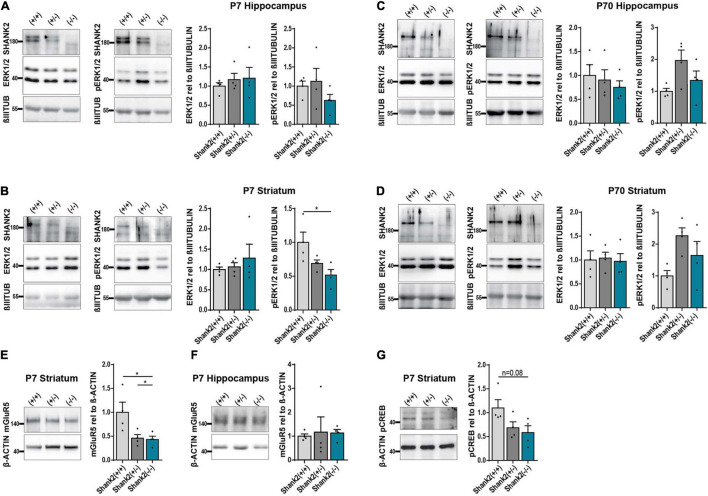
The ERK1/2 signaling pathway is changed in striatum of P7 but not P70 *Shank2*(–/–) mice. **(A)** SHANK2, ERK1/2, and phospho-ERK1/2 protein expressions relative to β-III-TUBULIN (*n* = 4) in hippocampus of P7 *Shank2*(+/+), *Shank2*(+/−), and *Shank2*(*–/–*) mice. **(B)** SHANK2, ERK1/2, and phospho-ERK1/2 protein expressions relative to β-III-TUBULIN (*n* = 4) in striatum of P7 *Shank2*(+/+), *Shank2*(+/−), and *Shank2*(–/–) mice. **(C)** SHANK2, ERK1/2, and phospho-ERK1/2 protein expressions relative to β-III-TUBULIN (*n* = 4) in hippocampus of P70 *Shank2*(+/+), *Shank2*(+/−), and *Shank2*(–/–) mice. **(D)** SHANK2, ERK1/2, and phospho-ERK1/2 protein expressions relative to β-III-TUBULIN (*n* = 4) in striatum of P70 *Shank2*(+/+), *Shank2*(+/−), and *Shank2*(–/–) mice. **(E)** mGluR5 protein expression relative to β-ACTIN (*n* = 4) in striatum of P7 *Shank2*(+/+), *Shank2*(+/−), and *Shank2*(–/–) mice. **(F)** mGluR5 protein expression relative to β-ACTIN (*n* = 4) in hippocampus of P7 *Shank2*(+/+), *Shank2*(+/−), and *Shank2*(–/–) mice. **(G)** phospho-CREB protein expression relative to β-ACTIN (*n* = 4) in striatum of P7 *Shank2*(+/+), *Shank2*(+/−), and *Shank2*(–/–) mice. βIIITUB: β-III-TUBULIN. Data were normalized to mean of *Shank2*(+/+). Mean ± SEM, one-way ANOVA, followed by Tukey’s *post hoc* analysis. Significance levels were set to *p ≤ 0.05.

To confirm that SHANK2 deficiency affects ERK1/2 signaling in general, we performed Western Blot analysis of 7-day-(P7)-old *Shank2*(+/+), *Shank2*(+/−), and *Shank2*(−/−) mice. Hippocampal tissue lysates showed no changes in ERK1/2 or phospho-ERK1/2 expression (Figure 6A), but, in striatum, (Figure 6B) phospho-ERK1/2 was significantly decreased in *Shank2*(−/−) mice, while total ERK1/2 stayed constant. Interestingly, none of these changes were observed in 70-day-(P70)-old adult mice (Figures 6C,D), indicating a neurodevelopmental dysregulation of the ERK1/2 pathway in striatum. Comparable to the hiPSC neuron data, AKT and phospho-AKT (Supplementary Figure 4G) were not altered in P7 striatum. Completing the picture, mGluR5 was also significantly decreased in P7 striatum (Figure 6E), but not in hippocampus (Figure 6F). P7 striatum also showed a clear trend for decreased phospho-CREB (Figure 6G). These data support the hypothesis of developmental ERK1/2 dysregulation under *SHANK2* deficiency during a specific window during neurogenesis that differentially affects specific brain regions.

## Discussion

In this study, we generated hiPSCs and derived neurons of a *SHANK2* deletion patient and two related controls. Molecular implications of *SHANK2* deletion have been studied in mouse models (Schmeisser et al., 2012; Won et al., 2012) and hiPSCs and derived neurons (Zaslavsky et al., 2019). hiPSCs offer the unique possibility to study human-derived cells that harbor a patient-specific “real-life mutation.” We showed that a heterozygous *SHANK2* deletion leads to a variety of alterations during early development and to profound alterations in the MAPK ERK1/2 signaling pathway. More mature cells manifested with an excitatory-inhibitory imbalance. Due to the neurodevelopmental emergence of ASD, we hypothesize that these early changes during neurodevelopment could contribute to the pathophysiology and synaptic changes seen in ASD.

Even though we analyzed one patient cell line, we were still able to translate our results to SHANK2 deficiency in general, since, firstly, we analyzed two related controls with the same genetic background than the patient. This gives strong genetic evidence that the changes we observed are, indeed, deriving from the *SHANK2* deletion. Secondly, we confirmed our findings in *Shank2*(−/−) mice, not only supporting our hiPSC data but also giving evidence for the observed alterations *in vivo*.

We found a profound dysregulation of the ERK1/2 signaling pathway in both young and mature neurons of the *SHANK2* deletion patient and in young *Shank2*(−/−) mice. Trying to integrate the human d21 and d42 neurons developed *in vitro* and the P7 and P70 mice into one shared developmental timeline, the P7 mice are considered more mature than the oldest hiPSC-derived neurons (Quadrato et al., 2017). This leads to a pattern of altered ERK1/2 signaling in all developmental timepoints P7 and younger. We, therefore, conclude that SHANK2 deficiency leads to a transient neurodevelopmental dysregulation of ERK1/2 signaling, that, in P70 animals, is not directly observed anymore. Nevertheless, as reported elsewhere (Schmeisser et al., 2012), these P70 animals show behavioral and molecular phenotypes that might, at least in part, be caused by the neurodevelopmental dysregulation of the ERK1/2 pathway. Our findings can help in tracing back the origin of these alterations and raise a better understanding of how neurodevelopment impacts on the mature brain.

Phospho-CREB, one of the main transcription factors activated by the ERK1/2 pathway, is important for a variety of cellular functions, including neuronal survival, cell proliferation and differentiation, and neuronal plasticity (Wang et al., 2018). The alterations we observed in the hiPSC-derived neurons (proliferation, apoptosis, and synaptic composition) could all be attributed to this disturbance of the ERK1/2-CREB axis. Consistently, mGluR5, phospho-ERK1/2, and phospho-CREB were also reduced in striatum of P7 *Shank2*(−/−) mice. Therefore, we claim that ERK1/2 and phospho-CREB can be regarded as central players in the pathophysiology of *SHANK2* mutations. CREB dysregulation is observed in various psychiatric disorders, including schizophrenia (McGirr et al., 2017) and ASD (Lyu et al., 2016), among others. In addition, decreased phospho-CREB levels have been observed in a valproic acid-induced ASD model in rats (Luhach et al., 2021), and various CREB-dependent genes have been found altered in an ASD hiPSC model system (Pasca et al., 2011). As summarized comprehensively by Wang et al. (2018), CREB as a modulator of neuronal plasticity is receiving increasing attention in ASD research, since, for example, mice deficient in certain CREB isoforms display an ASD-like behavior.

During early neuronal development, we found a reduced growth cone area, reduced apoptosis, increased cell proliferation, and an increased soma size in *SHANK2* deletion neurons. Increased proliferation and reduced apoptosis have already been shown in SH-SY5Y cells with *SHANK2* mutations, mimicking early neuronal development (Unsicker et al., 2021). Unsicker et al. (2021) linked these findings to increased phospho-AKT expression, which was not observed in our study. However, Unsicker et al. (2021) used a less complex model system and, in addition, only observed increased phospho-AKT levels in the biallelic *SHANK2* mutation, possibly triggering certain signaling pathways stronger than our heterozygous deletion. SHANK2 is known to localize to the growth cone (Du et al., 1998), and the reduction of growth cone area we observed under *SHANK2* deletion is nearly identical to the effect on growth cones seen by *SHANK3* knockdown (Huang et al., 2019). Changes in neuronal soma size seem to be an important morphological hallmark recurring in ASD (Wegiel et al., 2015) and have already been described in *SHANK3*-mutated hiPSC-derived neurons (Kathuria et al., 2017; Huang et al., 2019).

Taking a step back and transferring these cellular changes on total brain, increased proliferation, decreased apoptosis, and increased soma size are likely to result in macrocephaly. Interestingly, macrocephaly has been associated with SHANK3 mutations and Phelan-McDermid syndrome (PMDS) (Cochoy et al., 2015; Costales and Kolevzon, 2015). Unfortunately, assessment of macrocephaly is not mentioned in the records of our *SHANK2* deletion patient (Leblond et al., 2014). These findings emphasize to appreciate ASD as neurodevelopmental disorder, where subtle changes during development can severely impact molecular mechanisms and clinical phenotypes.

Besides the changes we found in young neurons, we observed synaptic changes in mature neurons of the *SHANK2* deletion patient. In synaptic immunostainings, we found significantly decreased mGluR5 and increased vGluT1, GluA2, and SHANK3 levels. mGluR5 binds to SHANK2 *via* Homer1b/c (Tu et al., 1999); thus, loss of postsynaptic SHANK2 could, indeed, lead to reduced postsynaptic mGluR5 levels. In support of our data, abnormal mGluR5 expression levels and function have been found in syndromic and non-syndromic forms of intellectual disability and ASD [Fragile X Syndrome (Bear et al., 2004), macrocephaly associated autism (Takeuchi et al., 2013), Rett Syndrome (Tao et al., 2016), and PMDS (Verpelli et al., 2011; Wang et al., 2016; Vicidomini et al., 2017)]. Reduced mGluR5 levels and reduced mGluR5-mediated long-term depression (LTD) were also described after the *SHANK3* knockdown in hippocampal neurons (Verpelli et al., 2011). Vicidomini et al. (2017) suggested that altered mGluR5 signaling could be one of the most common impaired signaling pathways in the *SHANK3* mutant brain after revealing altered mGluR5 function in striatum and cortex of *Shank3*Δ*11*(−/−) mice. mGluR5 is an important mediator in LTD, activation of ERK/MAPK-induced AMPAR mobilization and internalization [Reviewed in Gladding et al. (2009)]. Thus, reduced mGluR5 levels could possibly be responsible for increased synaptic GluA2 levels we observed in our *SHANK2*-deleted neurons. In support of our data, Verpelli and colleagues activated mGluR5 and found a decrease in number of synaptic GluA1 puncta in control cells that were not seen in *SHANK3* knockdown neurons (Verpelli et al., 2011). Consistently, increased GluA2 levels have been found in striatum of *Shank2e7*(−/−) mice (Schmeisser et al., 2012). Interestingly, striatal SHANK3 expression was increased in this mouse model, in accordance with our findings of increased synaptic SHANK3 expression in the *SHANK2* deletion patient. Thus, similar molecular pathways are altered in mouse and human *SHANK2* deletion systems. Besides regulation of mGluR5 through SHANK3, also SHANK2 was reported to modulate mGluR5 function. SHANK2 binds phospholipase C (PLC) that is activated G-protein mediated after mGluR5 activation (Hwang et al., 2005). Loss of SHANK2 thus provides less PLC and abolishes its downstream signaling that includes increase of intracellular calcium, MAPK activation, and AKT-mTOR signaling (Hwang et al., 2005). Furthermore, the central role of mGluR5 in the pathophysiology of SHANK2 deficiency is supported by a study in *Shank2*(−/−) mice where animals have been treated with CDPPB, a positive allosteric modulator of mGluR5 (Won et al., 2012). This treatment enhanced the diminished social interaction of these mice. However, the authors attributed the effects to normalized NMDA receptor function through mGluR5 activation. The GluN1 expression analyzed in our study did not show differences between parents and patients, demonstrating differences depending on the model systems used. Regarding increased vGluT1 levels, concordant reduction of mGluR5 and increase of vGluT1 levels were found in aged animals suffering from memory deficits (Menard et al., 2015). Intellectual disability is a hallmark of SHANK-related ASD (Leblond et al., 2014) and present in the *SHANK2* deletion patient of this study (Leblond et al., 2014), pointing toward common trajectories responsible for memory deficits and cognitive function in ASD.

Integrating these complex changes at excitatory glutamatergic synapses in a picture of “the” SHANK2-deficient synapse, we postulate increased expression of presynaptic vGluT1 and postsynaptic GluA2, suggesting increased synaptic transmission, while GluN1 expression was found unchanged. However, this seems to be opposed by decreased mGluR5 expression and its downstream ERK1/2 axis. We did not observe changes in synaptic proteins of inhibitory synapses, raising a possible imbalance between excitatory and inhibitory signaling. We can only speculate on what is happening first and triggering a second event. Nevertheless, given the abovementioned mutual relation of AMPA receptors, mGluRs, and vGluT1, they critically depend on one another. Even though increased synaptic transmission might be present, the effects of these synaptic alterations on the cell itself lead to decreased ERK1/2 signaling, impacting on core mechanisms of the cell, including proliferation and apoptosis.

Comparing our *SHANK2* deletion hiPSC line to previously published studies, Chen et al. (2020) investigated the effects of *SHANK2* knockdown on early neuronal development by infecting hiPSC-derived neurons with a shSHANK2 lentivirus (Chen et al., 2020). They found the dendritic length and arborizations of shSHANK2 vGLUT1 positive neurons to be shorter and fewer. In contrast, the only so-far published study using patient-derived hiPSC lines harboring a *SHANK2* deletion or mutation found an increase of dendritic length and increased synaptic numbers in *SHANK2* mutant cortical neurons (Zaslavsky et al., 2019). They conclude that lowered SHANK2 dosage increases the total synapse number resulting in neuronal hyperconnectivity, as shown by increases in dendrite length and complexity (Zaslavsky et al., 2019). In young *SHANK2*-mutated neurons, they found an enrichment of cell cycle genes, which is in line with our finding of increased proliferation in young neurons of the *SHANK2* deletion patient. However, in contrast to our findings, in older neurons with *SHANK2* mutations, the authors found elevated transcripts of mGluR1 and mGluR5, together with an enrichment of genes involved in synapse assembly and chemical synaptic transmission. Altogether, their *SHANK2*-mutated cell lines exhibited hyperconnectivity and a high expression of glutamate receptor signaling genes (Zaslavsky et al., 2019). Even though the deletion of our study and their study [Zaslavsky et al. (2019) used first a *SHANK2* nonsense mutation, second, a 66-kb deletion, and, third, a CRISPR/Cas9-generated homozygous knockout cell line] are all located in the *SHANK2* gene, differences between the cell lines might be explained by the neuronal cell types generated from the hiPSCs or by the postulated “multiple-hit model” for SHANK2-ASD (Leblond et al., 2012), claiming that additional, so-called modifier genes, might be altered in patients with SHANK2-ASD, which impact on both phenotypic and molecular alterations to be observed.

In summary, we generated hiPSC-derived neurons of a patient with *SHANK2* deletion and hypothesize that neurodevelopmental dysregulation contributes to synaptic changes in mature neurons. Our data support the hypothesis that an early dysregulation of the ERK1/2 signaling pathway might be commonly impaired in SHANK2-ASD.

## Data Availability Statement

The raw data supporting the conclusions of this article will be made available by the authors, without undue reservation.

## Ethics Statement

Informed consent was obtained from all human donors or their legal guardians prior to sampling. This study was approved by the Ethics Committee of Ulm University (proposal numbers 208/16 and 265/12) and was performed in accordance with institutional and national guidelines and regulations. Written informed consent to participate in this study was provided by the participants’ legal guardian/next of kin. All animal experiments were performed in compliance with the guidelines for the welfare of experimental animals issued by the Federal Government of Germany, National Institute of Health, or Max Planck Society. The experiments in this study were approved by the review board of the Land Baden-Württemberg, permit numbers O.103, 321/16, and 966/2016-PR, respectively. *Shank2*(−/−) mice (mus musculus) were generated as previously described (Schmeisser et al., 2012). Breeding was carried out as heterozygous breeding on a C57BL/6 background, animals were housed in standard laboratory conditions (average temperature of 22°C with food and water available *ad libitum*, dark/light cycle as 12/12 rhythm).

## Author Contributions

A-KL, MD, and TMB designed the project and experiments. A-KL, AP, and NS performed the experiments. VI analyzed the protein arrays. RD and TB provided patient/control hair roots and keratinocytes for the hiPSC lines. All authors contributed to the article and approved the submitted version.

## Conflict of Interest

The authors declare that the research was conducted in the absence of any commercial or financial relationships that could be construed as a potential conflict of interest.

## Publisher’s Note

All claims expressed in this article are solely those of the authors and do not necessarily represent those of their affiliated organizations, or those of the publisher, the editors and the reviewers. Any product that may be evaluated in this article, or claim that may be made by its manufacturer, is not guaranteed or endorsed by the publisher.

## Supplementary Material

Supplementary Figure 1 Characterization of hiPSC lines. **(A)** A scheme of hiPSC reprogramming. **(B)** Pluripotency staining for SOX2, OCT4, NANOG, TRA-1-81, TRA-1-60, and SSEA4. Scale bar = 50 μm. **(C)** Expression of pluripotency genes KLF4, SOX2, OCT4, and NANOG. Mean ± SEM. **(D)** Germline differentiation into mesoderm, endoderm ectoderm. Cells stained for α-ACTININ, alpha-fetoprotein (AFP), and β-III-TUBULIN. Scale bar = 50 μm. **(E)** Karyograms.

Supplementary Figure 2 Alterations in hiPSC and derived neurons. **(A)** hiPSCs stained for SHANK2. Scale bar = 50 μm. **(B,C)** Number of primary neurites in neurons d21 **(B)** and d42 **(C)**. Scale bar = 10 μm. **(D)** Caspase3 activity in colorimetric activity assay measured at 405 nm in neurons d21 and d42, *n* = 4. **(E)** hiPSCs stained for SHANK3. Scale bar = 50 μm. **(F–H)** *SHANK3* RNA and SHANK3 protein expression in hiPSCs **(F)**, neurons d21 **(G)**, and d42 **(H)**. Data were normalized to mean of parents if indicated. Mean ± SEM. Significance level was set to **p* ≤ 0.05.

Supplementary Figure 3 Expression of inhibitory and excitatory markers in hiPSC-derived neurons. **(A)** Analysis of total synaptic puncta intensity for SHANK2, SHANK3, HOMER1, mGluR5, GluA2, and GluN1. **(B)** Analysis of puncta per 30 μm of dendrite for SHANK2, SHANK3, HOMER1, mGluR5, GluA2, and GluN1. Mean ± SEM. **(C)** Analysis of total synaptic puncta intensity for vGluT1, vGAT, and BSN. **(D)** Analysis of puncta per 30 μm of dendrite for vGluT1, vGAT, and BSN. Mean ± SEM. **(E)** Whole neuron d42 stained against SHANK2, vGLUT1, and NFH. **(F)** Analysis of total synaptic puncta intensity for vGLuT1, co-localizing with SHANK2. **(G)** Analysis of puncta per 30 μm of dendrite for vGLuT1, co-localizing with SHANK2. **(H)** Total protein lysate of neurons d42 immunoblotted against GAD65, mGluR5, and GLUA2 relative to NFH. Data were normalized to mean of parents. Mean ± SEM. vGluT1, vesicular glutamate transporter 1; vGAT, vesicular GABA transporter; NFH, neurofilament heavy chain; BSN, bassoon; mGluR5, metabotropic glutamate receptor 5; GluA2, AMPA receptor subunit 2; GluN1, NMDA receptor subunit 1, GAD65, glutamic acid decarboxylase 65. Box plots show the median and the distribution of the minimum to the maximum data. One-way ANOVA followed by Tukey’s *post hoc* analysis. Significance levels were set to **p* ≤ 0.05, ***p* ≤ 0.01, ****p* ≤ 0.001, and *****p* ≤ 0.0001.

Supplementary Figure 4 ERK and AKT signaling in neurons and *Shank2*(−/−) mice. **(A)** Total protein lysate of neurons d21 immunoblotted against ERK1/2 and phospho-ERK1/2 relative to NFH. **(B)** Total protein lysate of neurons d21 immunoblotted against AKT and phospho-AKT relative to NFH. **(C)** Total protein lysate of neurons d42 immunoblotted against ERK1/2 and phospho-ERK1/2 relative to NFH. **(D)** Total protein lysate of neurons d42 immunoblotted against AKT and phospho-AKT relative to NFH. **(E)** Total protein lysate of neurons d21 immunoblotted against CREB and phospho-CREB relative to NFH. **(F)** Total protein lysate of neurons d42 immunoblotted against CREB and phospho-CREB relative to NFH. **(G)** AKT and phospho-AKT protein expression relative to β-ACTIN (*n* = 4) in striatum of P7 *Shank2*(+/+), *Shank2*(+/−), and *Shank2*(−/−) mice. Mean ± SEM, one-way ANOVA, followed by Tukey’s *post hoc* analysis. Significance levels were set to **p* ≤ 0.05, ***p* ≤ 0.01, ****p* ≤ 0.001, and *****p* ≤ 0.0001.
