# Supplementary figures and images for: SHANK2 Mutations Result in Dysregulation of the ERK1/2 Pathway in Human Induced Pluripotent Stem Cells-Derived Neurons and Shank2(−/−) Mice
Source: Front Mol Neurosci. 2021 Nov 26;14:773571. doi: 10.3389/fnmol.2021.773571 (PMC8662699; doi:10.3389/fnmol.2021.773571)

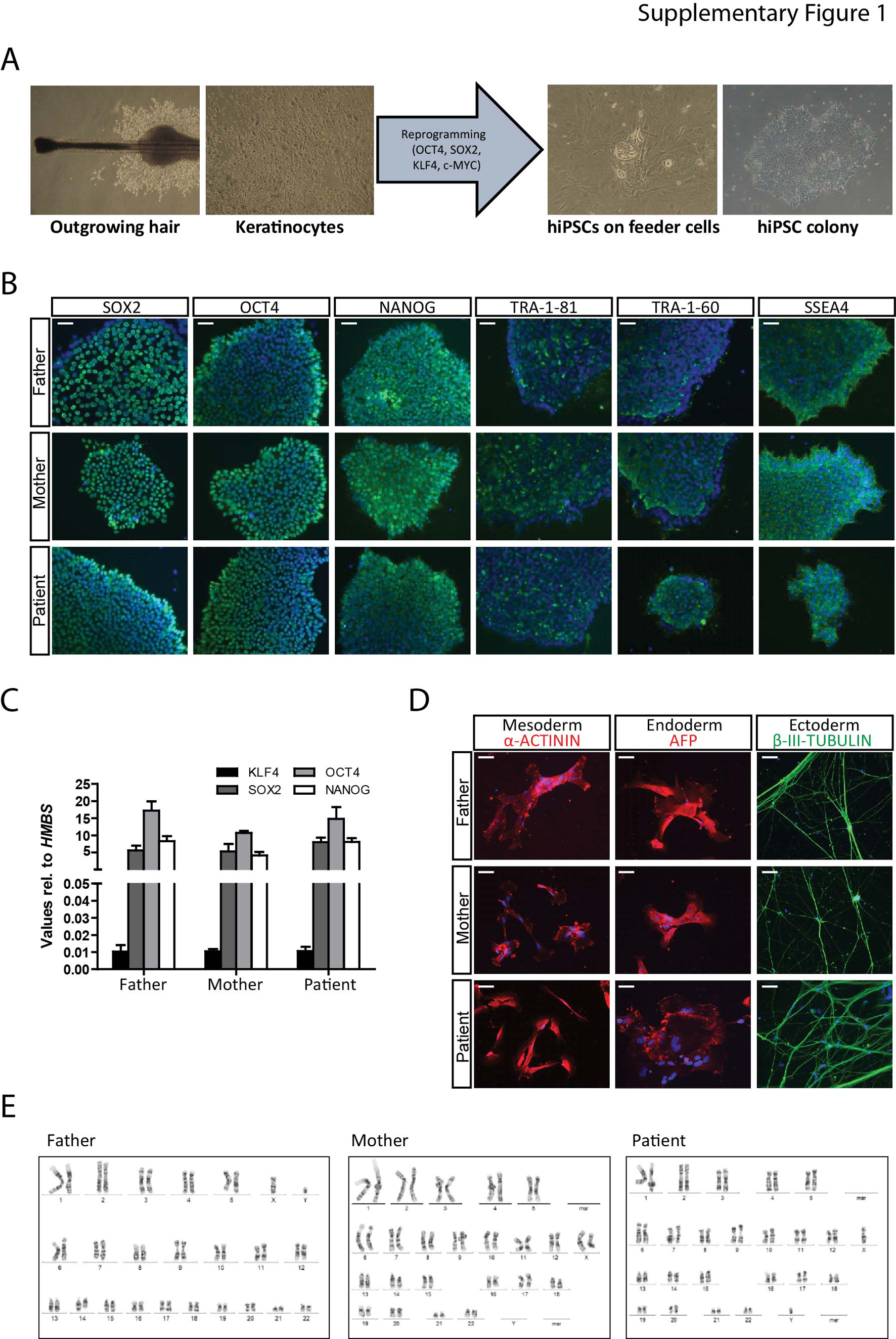

Supplement: Supplementary Figure 1 — Characterization of hiPSC lines. (A) A scheme of hiPSC reprogramming. (B) Pluripotency staining for SOX2, OCT4, NANOG, TRA-1-81, TRA-1-60, and SSEA4. Scale bar = 50 μm. (C) Expression of pluripotency genes KLF4, SOX2, OCT4, and NANOG. Mean ± SEM. (D) Germline differentiation into mesoderm, endoderm ectoderm. Cells stained for α-ACTININ, alpha-fetoprotein (AFP), and β-III-TUBULIN. Scale bar = 50 μm. (E) Karyograms. [file Image_1.JPEG]

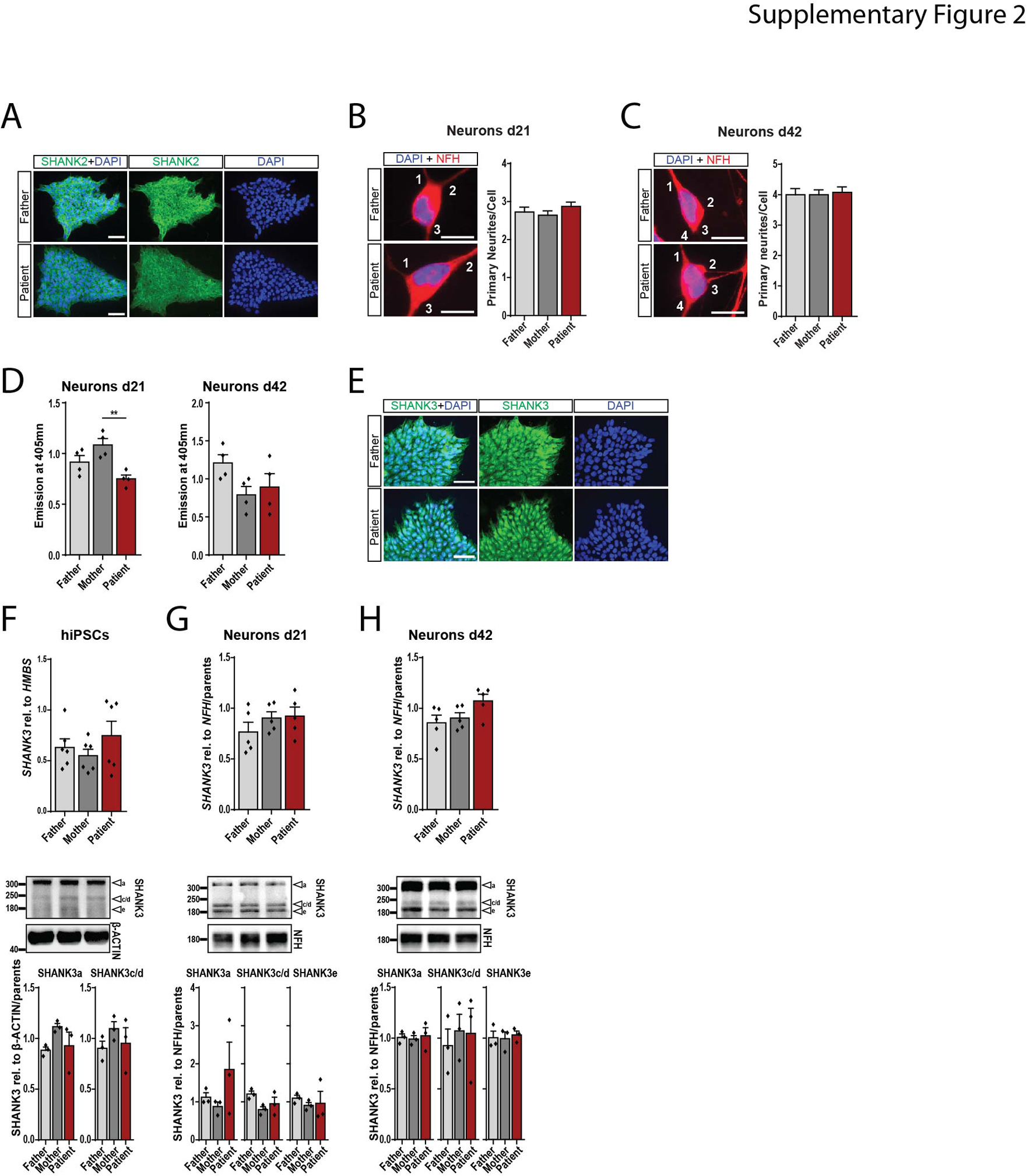

Supplement: Supplementary Figure 2 — Alterations in hiPSC and derived neurons. (A) hiPSCs stained for SHANK2. Scale bar = 50 μm. (B,C) Number of primary neurites in neurons d21 (B) and d42 (C). Scale bar = 10 μm. (D) Caspase3 activity in colorimetric activity assay measured at 405 nm in neurons d21 and d42, n = 4. (E) hiPSCs stained for SHANK3. Scale bar = 50 μm. (F–H) SHANK3 RNA and SHANK3 protein expression in hiPSCs (F), neurons d21 (G), and d42 (H). Data were normalized to mean of parents if indicated. Mean ± SEM. Significance level was set to *p ≤ 0.05. [file Image_2.JPEG]

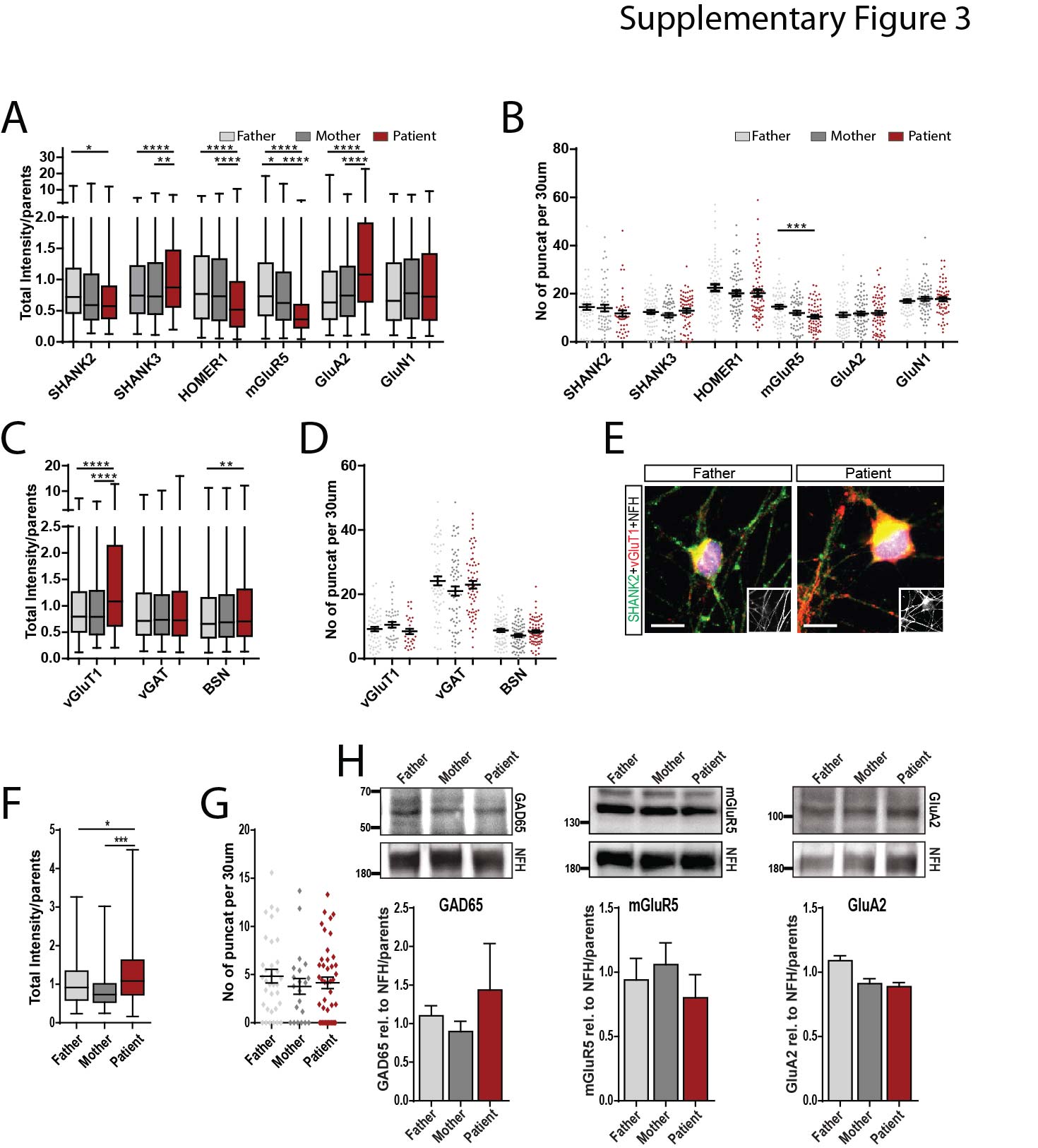

Supplement: Supplementary Figure 3 — Expression of inhibitory and excitatory markers in hiPSC-derived neurons. (A) Analysis of total synaptic puncta intensity for SHANK2, SHANK3, HOMER1, mGluR5, GluA2, and GluN1. (B) Analysis of puncta per 30 μm of dendrite for SHANK2, SHANK3, HOMER1, mGluR5, GluA2, and GluN1. Mean ± SEM. (C) Analysis of total synaptic puncta intensity for vGluT1, vGAT, and BSN. (D) Analysis of puncta per 30 μm of dendrite for vGluT1, vGAT, and BSN. Mean ± SEM. (E) Whole neuron d42 stained against SHANK2, vGLUT1, and NFH. (F) Analysis of total synaptic puncta intensity for vGLuT1, co-localizing with SHANK2. (G) Analysis of puncta per 30 μm of dendrite for vGLuT1, co-localizing with SHANK2. (H) Total protein lysate of neurons d42 immunoblotted against GAD65, mGluR5, and GLUA2 relative to NFH. Data were normalized to mean of parents. Mean ± SEM. vGluT1, vesicular glutamate transporter 1; vGAT, vesicular GABA transporter; NFH, neurofilament heavy chain; BSN, bassoon; mGluR5, metabotropic glutamate receptor 5; GluA2, AMPA receptor subunit 2; GluN1, NMDA receptor subunit 1, GAD65, glutamic acid decarboxylase 65. Box plots show the median and the distribution of the minimum to the maximum data. One-way ANOVA followed by Tukey’s post hoc analysis. Significance levels were set to *p ≤ 0.05, **p ≤ 0.01, ***p ≤ 0.001, and ****p ≤ 0.0001. [file Image_3.JPEG]

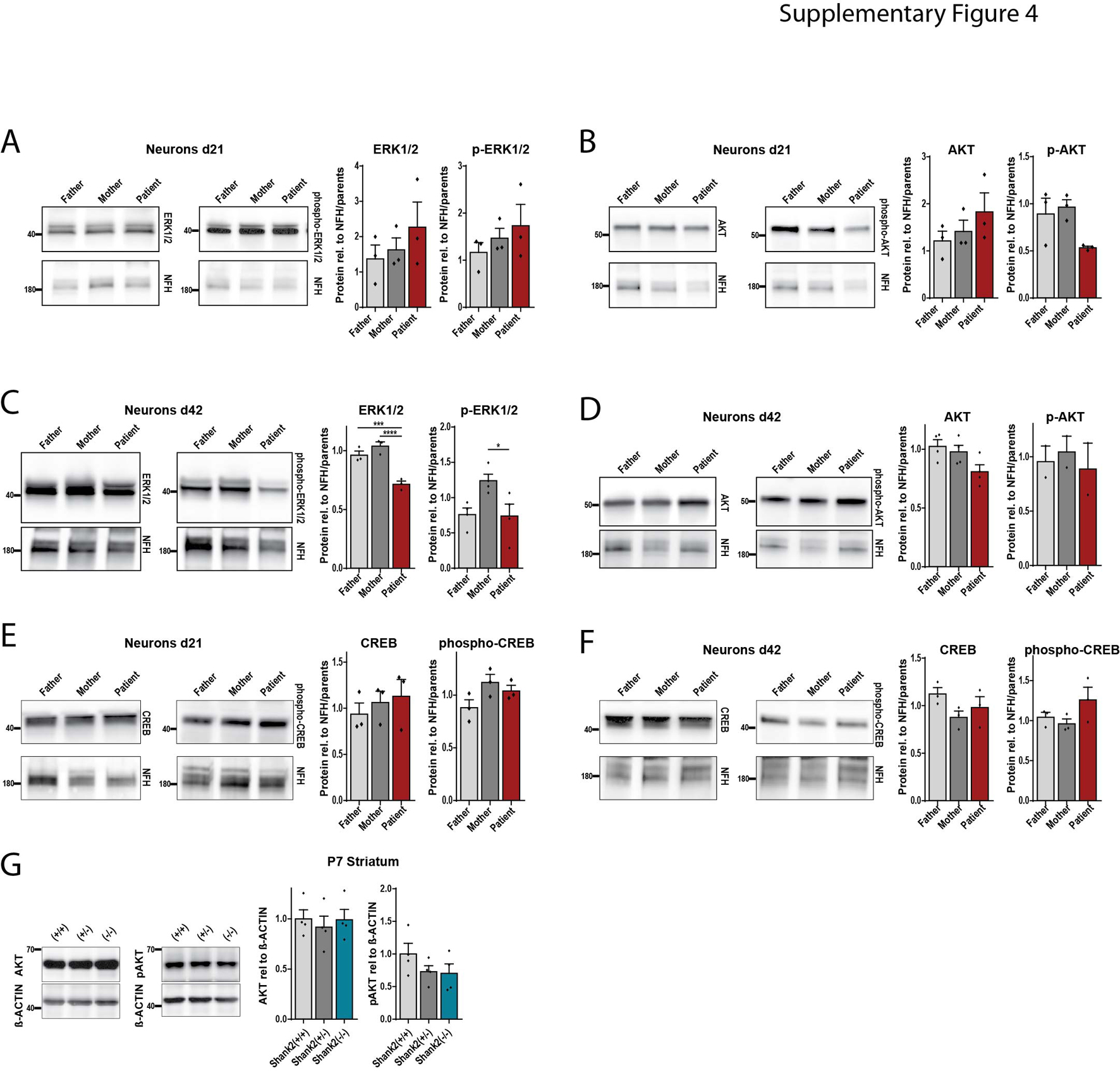

Supplement: Supplementary Figure 4 — ERK and AKT signaling in neurons and Shank2(−/−) mice. (A) Total protein lysate of neurons d21 immunoblotted against ERK1/2 and phospho-ERK1/2 relative to NFH. (B) Total protein lysate of neurons d21 immunoblotted against AKT and phospho-AKT relative to NFH. (C) Total protein lysate of neurons d42 immunoblotted against ERK1/2 and phospho-ERK1/2 relative to NFH. (D) Total protein lysate of neurons d42 immunoblotted against AKT and phospho-AKT relative to NFH. (E) Total protein lysate of neurons d21 immunoblotted against CREB and phospho-CREB relative to NFH. (F) Total protein lysate of neurons d42 immunoblotted against CREB and phospho-CREB relative to NFH. (G) AKT and phospho-AKT protein expression relative to β-ACTIN (n = 4) in striatum of P7 Shank2(+/+), Shank2(+/−), and Shank2(−/−) mice. Mean ± SEM, one-way ANOVA, followed by Tukey’s post hoc analysis. Significance levels were set to *p ≤ 0.05, **p ≤ 0.01, ***p ≤ 0.001, and ****p ≤ 0.0001. [file Image_4.JPEG]
